# Raise and characterization of a bread wheat hybrid line (Tulaykovskaya 10 × Saratovskaya 29) with chromosome 6Agi2 introgressed from Thinopyrum intermedium

**DOI:** 10.18699/VJ21.080

**Published:** 2021-11

**Authors:** Yu.N. Ivanova, K.K. Rosenfread, A.I. Stasyuk, E.S. Skolotneva, O.G. Silkova

**Affiliations:** Institute of Cytology and Genetics of the Siberian Branch of the Russian Academy of Sciences, Novosibirsk, Russia; Novosibirsk State Agrarian University, Novosibirsk, Russia; Kurchatov Genomic Center of ICG SB RAS, Novosibirsk, Russia; Institute of Cytology and Genetics of the Siberian Branch of the Russian Academy of Sciences, Novosibirsk, Russia; Institute of Cytology and Genetics of the Siberian Branch of the Russian Academy of Sciences, Novosibirsk, Russia

**Keywords:** alien introgression, chromosome substitution, GISH, molecular analysis, stem rust, brown rust, yellow rust, Thinopyrum intermedium, bread wheat, чужеродная интрогрессия, замещение хромосом, GISH, молекулярный анализ, стеблевая ржавчина, бурая ржавчина, желтая ржавчина, Thinopyrum intermedium, мягкая пшеница

## Abstract

Wheatgrass Thinopyrum intermedium is a source of agronomically valuable traits for common wheat. Partial wheat–wheatgrass amphidiploids and lines with wheatgrass chromosome substitutions are extensively used as intermediates in breeding programs. Line Agis 1 (6Agi2/6D) is present in the cultivar Tulaykovskaya 10 pedigree. Wheatgrass chromosome 6Agi2 carries multiple resistance to fungal diseases in various ecogeographical zones. In this work, we studied the transfer of chromosome 6Agi2 in hybrid populations Saratovskaya 29 × skaya 10 (S29 × T10) and Tulaykovskaya 10 × Saratovskaya 29 (T10 × S29). Chromosome 6Agi2 was identif ied by PCR with chromosome-specif ic primers and by genomic in situ hybridization (GISH). According to molecular data, 6Agi2 was transmitted to nearly half of the plants tested in the F2 and F3 generations. A new breeding line 49-14 (2n = 42) with chromosome pair 6Agi2 was isolated and characterized in T10 × S29 F5 by GISH. According to the results of our f ield experiment in 2020, the line had high productivity traits. The grain weights per plant (10.04 ± 0.93 g) and the number of grains per plant (259.36 ± 22.49) did not differ signif icantly from the parent varieties. The number of grains per spikelet in the main spike was signif icantly higher than in S29 ( p ≤ 0.001) or T10 ( p ≤ 0.05). Plants were characterized by the ability to set 3.77 ± 0.1 grains per spikelet, and this trait varied among individuals from 2.93 to 4.62. The grain protein content was 17.91 %, and the gluten content, 40.55 %. According to the screening for fungal disease resistance carried out in the f ield in 2018 and 2020, chromosome 6Agi2 makes plants retain immunity to the West Siberian population of brown rust and to dominant races of stem rust. It also provides medium resistant and medium susceptible types of response to yellow rust. The possibility of using lines/varieties of bread wheat with wheatgrass chromosomes 6Agi2 in breeding in order to increase protein content in the grain, to confer resistance to leaf diseases on plants and to create multif lowered forms is discussed.

## Abstract (ru)

Пырей промежуточный Thinopyrum intermedium является источником агрономически ценных признаков для мягкой пшеницы, для передачи которых используют частичные пшенично-пырейные амфидиплоиды и линии с замещением хромосомами пырея. С использованием линии Агис 1 создан сорт яровой мягкой пшеницы Тулайковская 5, который входит в родословную сорта Тулайковская 10. В геноме сорта хромосома пшеницы 6D замещена хромосомой пырея 6Agi2, несущей комплексную устойчивость к грибным заболеваниям в различных эколого-географических зонах. В данной работе изучен характер передачи хромосомы пырея 6Agi2 в гибридных популяциях сортов Саратовская 29 × Тулайковская 10 (С29 × Т10) и Тулайковская 10 × Саратовская 29 (Т10 × С29). Хромосома пырея 6Agi2 идентифицирована с помощью хромосомоспецифичных праймеров и методом геномной in situ гибридизации. Согласно молекулярному анализу, хромосома 6Agi2 передавалась почти половине изученных растений в F2 и F3 поколениях. В F5 поколении Т10 × С29 с помощью GISH выделена и охарактеризована новая селекционная линия 49-14 (2n = 42) с парой хромосом 6Agi2. По результатам эксперимента в полевых условиях 2020 г. линия имела высокие показатели продуктивности. Масса зерен с растения (10.04 ± 0.93 г) и число зерен с растения (259.36 ± 22.49) достоверно не отличались от родительских сортов. Число зерен на колосок в главном колосе у линии 49-14 было достоверно выше, чем у сортов С29 (при р ≤ 0.001) и Т10 (при р ≤ 0.05). Растения характеризовались способностью завязывать 3.77 ± 0.1 зерна на колосок, размах изменчивости признака варьировал от 2.93 до 4.62 у индивидуальных растений. Содержание белка в зерне составило 17.91 %, клейковины – 40.55 %. Согласно скринингу на устойчивость к грибным болезням, проведенному в полевых условиях 2018 и 2020 гг., хромосома 6Agi2 сохраняет у растений иммунность к западносибирской популяции бурой ржавчины и к доминантным расам стеблевой ржавчины, а также обеспечивает средний устойчивый и средний восприимчивый типы реакции к возбудителям желтой ржавчины. Обсуждается возможность использования линий/сортов мягкой пшеницы, несущих хромосомы пырея 6Agi2, в селекции на увеличение содержания белка в зерне, на устойчивость к листостебельным заболеваниям и на создание многоцветковых форм.

## Introduction

Wild perennial common wheat relatives of the Thinopyrum genus are broadly polymorphic. They can be sources of commercially valuable traits: resistance to fungal and viral diseases (Friebe et al., 1996; Li H., Wang, 2009; Krupin et al., 2013, 2019; Davoyan et al., 2015; Leonova, 2018), tolerance of saline soils and drought, and high protein contents in the grain (Tsitsin, 1954; Upelniek et al., 2012). The Thinopyrum genus includes about 20 species of different ploidies: diploids, allotetraploids, allohexaploids, octoploids, and decaploids (Wang R., 2011). The genetic pools of two species are in the greatest use: elongate wheatgrass Th. elongatum (Agropyron elongatum) and intermediate wheatgrass Th. intermedium (Ag. glaucum). They became donors of genes for resistance to pests: Lr19, Lr24, Lr29, and Lr38 to brown rust; Sr24, Sr25, Sr26, Sr43, and Sr44 to stem rust; Pm40 and Pm43 to powdery mildew; Bdv2 to barley yellow dwarf virus; and Wsm1 to wheat streak mosaic virus (Li H., Wang, 2009).

Viable wheat–wheatgrass hybrids were first obtained by N.V. Tsitsin in 1930–1933. He crossed diploid, tetraploid, and hexaploid wheats to Ag. elongatum and Ag. glaucum (Tsitsin, 1954) and obtained octoploid forms of perennial and ratooning wheats known as intermediate wheat–wheatgrass hybrids, IWWHs (Tsitsin, 1954; Upelniek et al., 2012). Experiments on wheat hybridization to plants of the Thinopyrum genus were also carried out in the United States, Germany, Canada, and China. Various hybrid forms were obtained and annotated: partial amphiploids; highprotein addition, substitution, and translocation lines and forms resistant to barley yellow dwarf virus, wheat streak mosaic virus, powdery mildew, yellow rust, brown rust, and stem rust (Friebe et al., 1996; Fedak, Han, 2005; Li H., Wang, 2009; Chang et al., 2010; Hu L. et al., 2011; Fu et al., 2012; Zeng J. et al., 2013; Bao et al., 2014; Zheng et al., 2014; Danilova et al., 2017; Li D. et al., 2018).

Partial wheat–wheatgrass amphidiploids are used internationally for transferring valuable traits to common wheat (Jiang et al., 1993; Fedak, Han, 2005). In Russia

two groups of common wheat cultivars resistant to fungal pests have been raised via IWWHs at the Agricultural Research Institute of the South-East and the Samara Research Institute of Agriculture. In their genomes, wheat chromosome 6D is replaced by chromosome 6Agi from wheatgrass Th. intermedium. Chromosomes 6Agi1 and 6Agi2 are not identical, as they show different C banding patterns in Giemsa staining (Sibikeev et al., 2017). In the former case, 6Agi1 was inherited from substitution line S29-Agro139-M2-2, obtained by crossing spring common wheat Saratovskaya 29 to IWWH 139, and from cv. Mnogoletka 2. Then wheatgrass addition chromosomes recombined with each other (Sibikeev et al., 2017). The cultivars raised in Samara inherited wheatgrass chromosome 6Agi2 from substitution line Agis 1, obtained by crossing S29 to IWWH 644 (Sinigovets, 1976, 1988).

Since 1984, when Tulaykovskaya 5 was enlisted to the State Register of Selection Achievements, varieties with wheatgrass chromosome introgression bred in Samara retain their resistance to brown rust and powdery mildew in various ecogeographical regions of Russia (Salina et al., 2015; Leonova et al., 2017). It has been shown that the Lr genes on chromosome 6Agi2 are not allelic to the genes Lr9, Lr19, Lr24, Lr29, or Lr47, and the type of response to inoculation with Puccinia triticina Eriks. isolates confirms their not being allelic to Lr19 or Lr38 (Sibikeev et al., 2017). Testing of F2 and F3 hybrids of susceptible varieties with Tulaykovskaya 10 for brown rust resistance shows that chromosome 6Agi2 houses a locus for resistance to the West Siberian brown rust race (Salina et al., 2015). However, the copy number of resistance genes on 6Agi2 is still unknown. The loci have not been mapped on the chromosome either.

Molecular and cytogenetic markers are designed for detection of wheatgrass genetic material in the common wheat genome (Han F. et al., 2004; Li G. et al., 2016; Cseh et al., 2019; Kroupin et al., 2019). There are molecular markers specific to the Th. intermedium genome: simple sequence repeats (SSRs) (Ayala-Navarrete et al., 2010), markers designed on the base of expressed sequences (ESTs) (Wang M.J. et al., 2010; Danilova et al., 2017), and specific locus amplified fragments (SLAFs) (Li G. et al., 2016). There are several RFLP (Zhang Z.Y. et al., 2001), SCAR (Liu et al., 2007), and ISSR (Zeng Z.-X. et al., 2008) markers for Pseudoroegneria spicata (St genome), designed for identification of particular chromosomes of the St genome. The correspondence of wheatgrass chromosomes to homoelogical common wheat groups is tested with unique gene markers based on PCR (PLUG markers) (Ishikawa et al., 2009; Hu L. et al., 2014) and SNP markers (Cseh et al., 2019; Ma et al., 2019). Salina et al. (2016) designed markers specific to the long and short arms of Th. intermedium chromosome 6Agi2

Varieties bred in Samara are used in Russian breeding programs (Martynov et al., 2016; Leonova, 2018). The goal of this work was to obtain breeding material with introgressed wheatgrass chromosome, test its commercially significant indices, and investigate the transfer of Th. intermedium chromosome 6Agi2 present in cv. Tulaykovskaya 10 by the example of a hybrid population with wheat cultivar Saratovskaya 29, which is a gold standard of grain quality. DNA markers specific to the long and short arms of 6Agi2 and genomic in situ hybridization (GISH) were used to identify the chromosome.

## Materials and methods

Plants. Experiments were conducted with spring common wheat varieties Saratovskaya 29 (S29) and Tulaykovskaya 10 (T10) and with their hybrids S29 × T10 (generations F2, F3) and T10 × S29 (generations F2–F6). The hybrid generations were obtained by self-pollination of F1 hybrids. Varieties S29 and T10 belong to the mid-season group. Saratovskaya 29 is highly susceptible to leaf diseases. Tulaykovskaya 10 is immune to brown leaf rust and mediumsensitive to powdery mildew (https://samniish.ru/pshenicamyagkaya- yarovaya-sort-tulajkovskaya-10.html).

Hybrids S29 × T10, generations F2 and F3, and T10 × S29, generations F2, F3 and F5, were grown in a hydroponic greenhouse of the Laboratory of Artificial Plant Growth, Institute of Cytology and Genetics, Novosibirsk, in the autumn of 2017 and in the springs of 2019 and 2020, respectively. The temperature schedule was 22 °C in the daytime and 16 °C at night. The light/dark schedule was 16:8 h. Hybrid generations T10 × S29 F4 and F6 were grown in the field in the Moshkovo raion of the Novosibirsk oblast in the summers of 2018 and 2020, respectively; locality coordinates 55.14° N and 83.63° E.

Fluorescence in situ hybridization (FISH). Mitotic chromosome slides for FISH were prepared as in Ivanova et al. (2019). Use was made of the Aegilops tauschii pAet6- 09 probe specific to chromosome centromeric repeats of rice, wheat, rye, and barley (Zhang P. et al., 2004) and wheatgrass genomic DNA isolated from Th. intermedium

plants. A DNA sample of the pAet6-09 repeat was kindly provided by Dr. A. Lukaszewski (University of California, Riverside, United States). All slides were examined under an Axio Imager M1 microscope (Karl Zeiss, Germany). Images were captured with a ProgRes MF camera (Meta Systems, Jenoptic) in the Shared Access Center for Microscopy Analysis of Biologic Objects, Siberian Branch of the RAS, and processed with Adobe Photoshop CS2.

Plant DNA isolation. DNA was isolated from young leaves of hybrids and control plants with a Genomic DNA Purification Kit (Thermo Scientific, No. K0512) according to manufacturer’s recommendations.

PCR analysis. DNA samples were analyzed with primers MF2/MR1r2 (amplicon size 347 bp) to the long arm of chromosome 6Agi2L of Th. intermedium, Te6HS476 (amplicon size 200 bp) to the short arm of chromosome 6Agi2S of Th. intermedium, and MF2/MR4 (amplicon size 328 bp) to the long arm of chromosome 6DL. The primers had been designed at the Laboratory of Plant Molecular Genetics and Cytogenetics, Institute of Cytology and Genetics (Salina et al., 2016). PCR was carried out in a Bio-Rad T-100 Thermal Cycler. The products were resolved in 1.5 % agarose gel with ethidium bromide and visualized with a Gel Doc XR+ gel documentation system (Bio-Rad, United States).

Assessment of commercially valuable traits. The T10 × S29 F4 progeny selected with molecular markers was tested for resistance to brown rust Puccinia triticina Eriks. and stem rust P. graminis Pers. in field experiments in 2018. The F6 progeny selected by molecular cytological analysis was tested for resistance to brown rust P. triticina Eriks., stem rust P. graminis Pers., and yellow rust P. glumarum Eriks. et Henn. in the field in 2020. The following parameters were recorded in generation F6 selected by molecular and cytological methods in the field in 2000: the sprouting–flowering interval, plant height, productive tillering, main spike length, number of spikelets in the main spike, number of grains in the main spike, grain weight of the main spike, number of grains per spikelet in the main spike, grain number per plant, grain weight per plant, 1000 grain weight, and contents of protein and gluten in the grain. Grains were sown on May 9, 2020, in plots of 70 cm in width, 15 grains per row, and 25-cm intervals between rows

The degree of injury by fungal pests was assessed according to the CIMMYT scale (Koyshybaev et al., 2014). The contents of protein and gluten were measured with an infrared OmegAnalyzer G (Bruins, Germany). The time from the mass-scale appearance of sprouts till the first appearance of yellow anthers in middle spikelets of spikes was taken to be the sprouting–flowering interval. Flowering dates were recorded in individual spikes. The significance of differences between two mean values of two samples was assessed by Student’s t test

## Results

Identification of wheatgrass chromosome 6Agi2 in generations F2–3 of the S29 × T10 and T10 × S29 hybrids with chromosome-specific primers

**Fig. 1.**
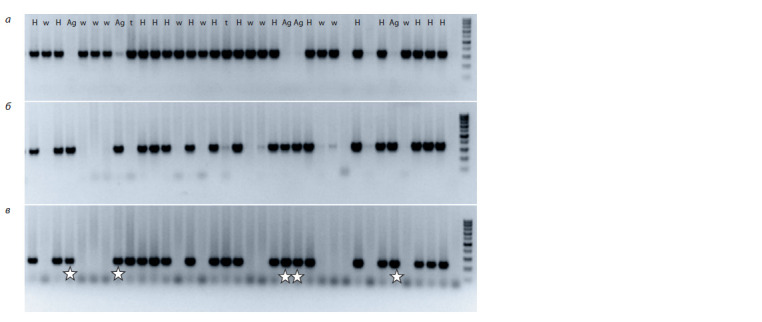
Electrophoretic image of the amplification of markers in F2 plants of the S29 × T10 hybrid. Markers: a, to the long arm of chromosome 6DL; b, to the short arm of 6Agi; c, to the long arm of 6Agi. Stars indicate plants with 6Agi2/6D substitution. Designations follow the text body and Table 1.

Chromosomes 6Agi2 of wheatgrass and 6D of wheat were present in the F1 of S29 × T10 and T10 × S29 in the univalent state. Therefore, their presence or absence in DNA samples from generation F2 was tested by PCR with primers specific to the wheatgrass chromosome. We tested 116 and 45 DNA samples from F2 S29 × T10 and T10 × S29,respectively, and found samples with the absence of amplification with two primer pairs for the short and long arms of chromosome 6Agi2 and with amplification of the marker to chromosome 6D. Thus, there were no 6Agi2/6D substitution in these samples, designated as wheat (w) type (Fig. 1, Table 1).

**Table 1.**
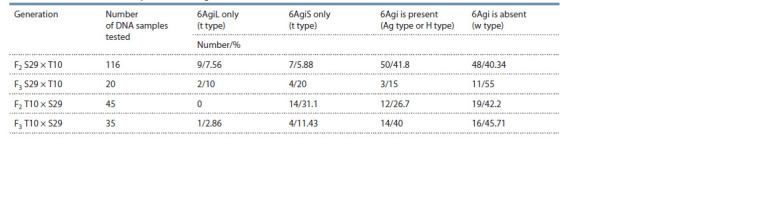
The presence or absence of chromosomes or chromosome arms in generations F2–3 of the S29 × T10 and T10 × S29 hybrids according to PCR data

The presence of chromosome 6D was also proven in samples with amplification of markers to either long or short arm, being indicative of the presence of telocentrics (t type; see Fig. 1, Table 1). Altogether, 12 telocentrics for the long arm and 29 telocentrics for the short arm were detected in samples of generations F2 and F3, and the ratio of telocentrics for the short and long arm depended significantly on the cross direction. Telocentrics for the long arm were very rare in the T10 × S29 cross.

The presence of amplification fragments with two markers to the short and long arms pointed to the presence of the whole chromosome 6Agi2. With regard to the presence or absence of chromosome 6D, we suggest either full 6Agi2/6D substitution (Ag type) or the heterozygous state of the chromosome in the samples (H type).

For further analysis, plants with amplification of markers to the short and long arm of the wheatgrass chromosome were selected.

**Fig. 2.**
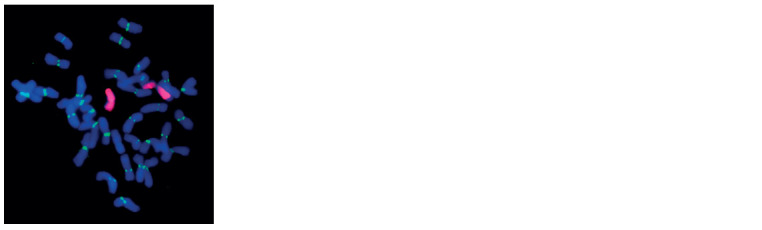
Chromosomes of generation F5 of T10 × S29 stained by GISH. The two wheatgrass chromosomes are stained red, and centromeric regions, green.

Karyotyping of generation F5 of T10 × S29 hybrids To verify the presence of one or two wheatgrass chromosomes in chromosome sets and to confirm stable inheritance of the substitution, we performed GISH of mitotic chromosomes at various self-pollination stages. The analysis of plants bearing substitutions according to PCR revealed 42 chromosomes, of which two were whole wheatgrass chromosomes (Fig. 2). Their long arms housed a large subtelomeric heterochromatin block, which is consistent with the locations of Giemsa C bands on chromosome 6Agi2 in Tulaykovskaya 10 (Sibikeev et al., 2017). The centromere-specific pAet6‑09 repeat located on wheatgrass chromosomes showed weak signals, to demonstrate the poor hybridization of the repeat to centromeric DNA of wheatgrass chromosomes.

In situ hybridization confirmed the stable inheritance of the 6Agi2/6D substitution through generations.

Commercially valuable traits in T10 × S29 generations F5 and F6

Tulaykovskaya 10 is present in the pedigrees of many modern common wheat varieties. Its use in the breeding of new forms is based on its locus for brown leaf rust resistance, mapped on wheatgrass chromosome 6Agi2. In spite of the replacement of chromosome 6D by alien chromosome 6Agi2, the variety shows high grain yield, drought tolerance, and good baking quality (https://samniish.ru/ pshenica-myagkaya-yarovaya-sort-tulajkovskaya-10.html).

Three lines were raised from F4 plants of T10 × S29 with identified wheatgrass chromosomes: 33-2, 34-1, and 35-45. Analysis of the performance of T10, S29, and T10 × S29 F5 lines grown in a hydroponic greenhouse showed that all the lines significantly outperformed T10 in all indices (Table 2). As compared to S29, the lines did not differ in productive tillering; lines 34-1 an 35-45 did not differ in grain number per plant or grain weight per plant; and in line 33-2, these indices were significantly lower. None of the lines outperformed S29 in 1000 grain weight; this index was significantly lower.

**Table 2.**
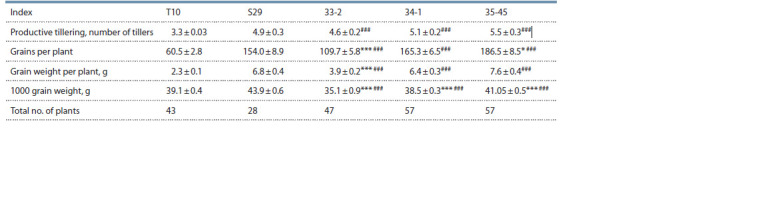
Performance indices in lines compared with varieties S29 and T10 (spring, 2019) Difference from S29 signif icant at * р ≤ 0.05; *** р ≤ 0.001. Difference from T10 signif icant at ### р ≤ 0.001.

We selected the most productive plants of generation F5 of line 35-45 to analyze performance indices and the duration of the sprouting–flowering interval in plants grown in the field in 2020. Thus, daughter line 49-14 was selected from the chosen segregating line 35-45.

Phenological observations revealed the shortest sprouting– flowering interval in line 49-14 (50.6 days), and in S29 and T10 it was one day longer. The flowering durations in the main spikes of individual plants were 11 days in 49-14, 10 days in T10, and 9 days in S29.

Comparison of performance indices in 49-14, S29, and T10 revealed no difference in main spike length, grain weight in the main spike, grain weight per plant, or grain number per plant (Table 3). Plants of line 49-14 were significantly taller than T10 but did not differ in height from S29. Productive tillering and main spike density in 49-14 showed significant ( p ≤ 0.05) differences from the cultivars. The number of grains in the main spike in 49-14 was significantly greater than in S29 ( р ≤ 0.001) or T10 ( р ≤ 0.05).

**Table 3.**
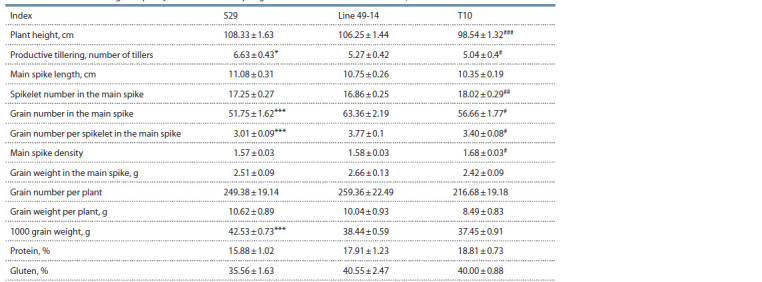
Performance and grain quality indices in the offspring of line 49-14 and cvs. T10 and S29, summer of 2020 Differences between S29 and 49-14 signif icant at * р ≤ 0.05; *** р ≤ 0.001. Differences between T10 and 49-14 signif icant at # р ≤ 0.05; ## р ≤ 0.01; ### р ≤ 0.001.

**Fig. 3:**
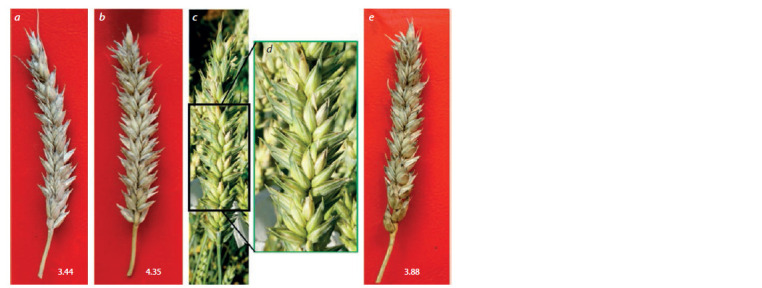
The main spikes of plants with the best numbers of grains per spikelet in the main spike: a, S29 (3.44); b, line 49-14 (4.35); c, spike of line 49-14 at the waxy maturity stage; d, the zoomed central spike portion in (c); e, T10 (3.88).

The number of grains per spikelet in the main spike of line 49-14 was significantly higher than in S29 ( p ≤ 0.001) or T10 ( p ≤ 0.05). Line 49-14 set 3.77 ± 0.1 grains per spikelet on the average, and this trait varied among individual plants from 2.93 to 4.62 (Fig. 3, see Table 3). The 1000 grain weights in line 49-14 and T10 were significantly ( р ≤ 0.001) lower than in S29.

Grain quality analysis showed that S29, T10, and 49- 14 had high contents of protein and gluten (see Table 3), characteristic of strong wheats. Grain quality in 49-14 was comparable with S29 and T10.

Screening of generations F4 and F6 of the T10 × S29 cross for resistance to fungal pathogens

The resistance of plants to brown rust and stem rust agents was tested in the field in 2018 and 2020. Field resistance to powdery mildew was not tested in those years, because weather conditions were unfavorable for the agent, as seen from the fact that the susceptible variety S29 was not injured.

**Fig. 4.**
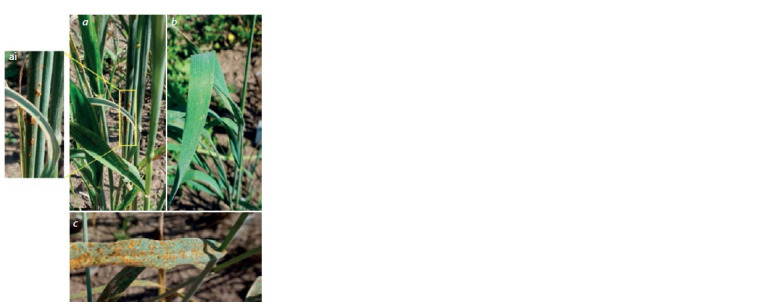
Absence of damage by brown rust from F4 T10 × S29 hybrids (a, b); brown rust damage of S29 (c); ai – culm damage by stem rust (zoomed). Photographed August 18, 2018.

In tests of the resistance to the Siberian population of the brown rust agent P. triticina conducted in 2018, S29 demonstrated the S (susceptibility) type of response, scoring 4 with about 100 % damage of leaf surface (Fig. 4, c). Tulaykovskaya 10 and F4 plants of T10 × S29 showed the immune type without P. triticina pustules (see Fig. 4, a, b).

The hybrids tested and parental varieties produced a specific response to stem rust. Plants of S29, T10, and F4 T10 × S29 showed generally the immune response except for a single case. One of the F4 plants showed a specific type of interaction with the pathogen: occasional uredial pustules without chlorosis (5S) (see Fig. 4, a, ai). In practice, the detected local but pronounced syndrome is interpreted as a sign of a rare virulent fungus race in the local population (Roelfs et al., 1992). As reported by Skolotneva et al. (2020), the stem rust population in the Novosibirsk oblast is highly heterogeneous, as it is formed by southern and western migrants.

**Fig. 5.**
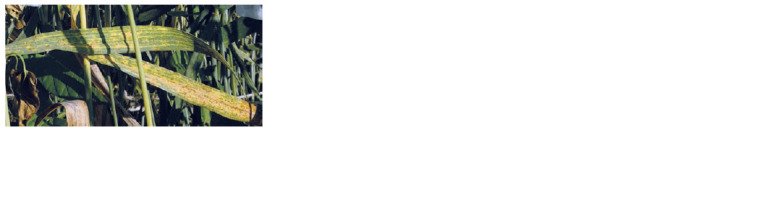
Leaves of S29 injured by yellow and brown rusts. Photographed August 2, 2020.

**Fig. 6.**
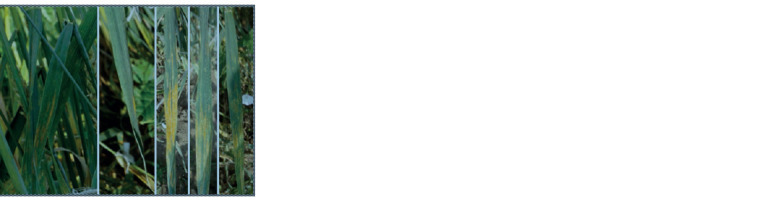
Resistance of 49-14 plants to brown rust and different degrees of damage by yellow rust. Photographed August 5, 2020.

No signs of fungal diseases were detected in plants of the cultivars and line 49-14 at the stages of tillering and flowering in the field in 2020. Tests for plant resistance to the brown rust population at the milky ripeness stage showed type S (susceptibility) response in S29 plants (Fig. 5), score 4 with about 100 % leaf damage, whereas T10 and 49-14 demonstrated the immune response with no P. triticina pustules (Fig. 6).

At the milky ripeness stage, on August 2–5, the start of damage of S29, T10, and 49-14 by the yellow rust agent P. striiformis was noted. The percentage of leaf area injury in S29 was 50 to 75 (see Fig. 5), corresponding to medium susceptibility (MS).

Plants of T10 and 49-14 showed medium resistance (MR) and medium susceptibility (MS) to the yellow rust agent. The percentage of leaf area injury was 5 to 40, with chlorotic zones (see Fig. 6).

No damage by stem rust was seen in plants of S29, T10, or 49-14 in the summer of 2020.

Thus, the results of screening for resistance to a variety of plant pathogens conducted in the field in different years indicate that chromosome 6Agi2 retains the immunity of plants to the West Siberian brown rust population and immunity to dominant stem rust races. It also supports the medium resistant and medium susceptible types of response to yellow rust agents.

## Discussion

Breeding line 49-14 (2n = 42) was isolated from generation F5 of intervarietal hybrids T10 × S29, with introgression of a pair of wheatgrass chromosomes 6Agi2. It shows high performance indices and immunity to West Siberian populations of brown rust agents. The response of 49-14 plants to the yellow rust agent varies from medium resistance to medium susceptibility, probably because of the difference in aggressiveness among the agent races. Stem rust injury was noted in only one plant and was interpreted as immunity to dominant stem rust races.

Previously, it was demonstrated that the genetic material of chromosome 6Agi2 in common wheat varieties Tulaykovskaya 5, Tulaykovskaya 10, Tulaykovskaya zolotistaya, Tulaykovskaya 100, and Volgouralskaya retains the resistance to brown rust populations typical of the Lower and Middle Volga regions, Central and Ural regions, and West Siberia (Plakhotnik et al., 2014; Salina et al., 2015; Leonova et al., 2017; Askhadullin et al., 2019). The damage of Tulaykovskaya 10 by brown rust in infection nurseries of the Central Chernozem region reached 22 %, and the variety was assigned to group II of epidemic resistance (moderately resistant ER II) (Zeleneva, 2019). In Tatarstan, the damage of Tulaykovskaya 10 by stem rust was assessed as 5–10 % on the average, and the damage by powdery mildew scored 6; the type of response to brown rust remained immune (Askhadullin et al., 2019). The susceptibility of T10 to the powdery mildew population of the West Siberian region was assessed as resistance. A genome-wide association search (GWAS) mapped the Pm6Agi2 gene on the long arm of wheatgrass chromosome 6Agi2, and this gene imparts resistance to the powdery mildew agent (Leonova, 2019). In experiments in the Middle Volga region, T10 showed immunity to brown rust and medium resistance (20 % injury) to stem rust, yellow rust, and powdery mildew (Syukov et al., 2016). Thus, T10 retains its immunity to brown rust populations in various ecogeographical regions. It is medium susceptible to stem and yellow rusts but shows diverse responses to the powdery mildew agent.

The substitution of wheatgrass chromosome 6Agi2 for 6D does not impair grain yield, grain quality, or drought tolerance (Filatova et al., 2010; Volkova et al., 2010), although in some cases of using T10 as a resistance gene donor, plants with lower productive tillering and 1000 grain weight appeared among the offspring with chromosome 6Agi2 (Stasyuk et al., 2017). The contents of protein and gluten in line 49-14 were about the same as in S29 or T10, corresponding to the grain quality of strong wheats (State Standard…, 2018, 2019). Line 49-14 lagged behind the parental varieties in productive tillering (S29), number of spikelets in the main spike (T10), and 1000 grain weight (S29). In spite of lower productive tillering, fewer spikelets in the main spike, and lower 1000 grain weight, the indices grain weight per plant and grain number per plant in line 49-14 did not differ significantly from the parental varieties owing to the significantly higher grain number per spikelet in the main spike of 49-14 than in S29 ( р ≤ 0.001) or T10 ( p ≤ 0.05). Plants of 49-14 set 3.77 ± 0.1 grains per spikelet, the range of variation in individual plants being 2.93–4.62, and up to 6 grains were set in spikelets of the middle spike part. Spikelets were fan-shaped (see Fig. 3). This shape is a specific sign of multiflowered spikelets in wheat (Martinek et al., 2005; Arbuzova et al., 2016).

Although common wheat has multiflowered spikelets, most of them set two or three grains. As the potential of forming more grains in wheat exceeds the actual yield by far, many studies are dedicated to seeking tools to control this process. The genetic and physiological grounds of breeding for more grains in spikes and spikelets and, ultimately, more grains per unit area are extensively investigated (Cui et al., 2012; Sreenivasulu, Schnurbusch, 2012; Arbuzova et al., 2016; Guo et al., 2016–2018; Bhusal et al., 2017; Philipp et al., 2018; Sukumaran et al., 2018; Wolde et al., 2019; Hu J. et al., 2020). Analysis of the reproductive developmental stages of spikes, spikelets, florets, and grains, as well as of their genetic regulation, is the best way to understand the formation of the trait ‘grain number and spike fertility’. The ‘grain number per spikelet’ trait depends on the initiation of floret primordia, then on floret survival at the next stage, and then on their efficient pollination. Normally, up to 12 floret primordia form at the white anther stage, but later up to 60 % of the florets may remain underdeveloped (Guo et al., 2016, 2017). This applies especially to apical (uppermost) florets of a spikelet. As reported by Kuperman (1969), the growth rates of the two lowest and upper floret apices are nonuniform at organogenesis stage V; a spikelet may have up to five, less often, to seven florets. Lower florets very quickly form primordia of generative organs, stamens, and the pistil. A delay in organ formation is observed in the third and, particularly, fourth, fifth, and subsequent florets. Pistils most often remain underdeveloped in the uppermost florets. Chromosomes 4A, 5A, 6A, 7A, 2B, 5B, 7B, and 7D bear QTLs responsible for the trait ‘number of floret primordia per spikelet’ (Guo et al., 2017). Also, the correlation and cluster analyses performed in the same study infer that the number of grains per spikelet does not depend on the maximum number of floret primordia per spikelet (Guo et al., 2017). Hence, the number of grains in a spikelet is determined by the fertility of each floret (Kuperman, 1969; Sreenivasulu, Schnurbusch, 2012).

A QTL responsible for greater numbers of grains per spikelet was detected on the long arm of chromosome 2A in GWAS of European common wheat varieties (Guo et al., 2017). Further studies of this locus mapped the Grain Number Increase 1 (GNI1) gene, encoding a transcription factor with the HDZip1 homeodomain. Its mutation contributes much to greater numbers of fertile florets due to upper florets of the spikelet (Sakuma et al., 2019). Supposedly, GNI1 was formed by gene duplication in wheat evolution, and its mutations were selected in domestication, as they increased the number of fertile florets, and, consequently, grains. Transcription factor ARGONAUTE1d (AGO1d ) also affects the grain number in the spikes of common and durum wheats (Feng et al., 2017). AGO1d is important for the development of anthers and pollen at early developmental stages of wheat. Its malfunction shortens the spike, reduces anther size, decreases pollen fertility, and thereby decreases the number of grains in the spike (Feng et al., 2017).

The manifestation of traits in a plant is cumulatively affected by the genotype, ambient conditions, and farming techniques. All these factors greatly influence quantitative traits, including yield components (Piskarev et al., 2016; Stasyuk et al., 2017). The day/night regime and solar spectrum are particularly important ambient factors at organogenesis stages V and VI (Kuperman, 1969). Lower intensities of the red and infrared radiation reduce the number of fertile florets, number of grains per plant, and 1000 grain weight (Ugarte et al., 2010). The combinations of environmental factors required for each developmental stage stem from the conditions under which the species, varieties, and cultivars formed. With regard to their physiological developmental features, cultivars S29 and T10 belong to the Volga steppe and forest-steppe agroecological groups, respectively, or morphophysiological type II (Kuperman, 1969) (https://samniish.ru/yarovaya_myagkaya_ pshenica.html). Such varieties utilize mainly winter and early spring precipitation in regions with water shortage in the second half of summer; that is, they are tolerant of summer drought. Cultivars bred in West Siberia belong to morphophysiological type V. The ecotype of Siberian forest-steppe wheats is determined by the climate: cold and dry April, May, and the first half of June; relatively ample precipitation in the second half of summer (July), and cold temperatures in August. The delay in organogenesis stage V allows much better use of late summer precipitation for the formation of large spikes and multiflowered spikelets.

Owing to developmental physiological features and high drought tolerance, varieties of morphophysiological type II can be grown in steppe and forest-steppe regions of West Siberia (Kuperman, 1969). Thus, the genotypes of S29 and T10 are environmentally flexible. In the climate of West Siberian forest-steppe, they synchronize the metameric growth of spikelets to develop four, five, or more normal florets in a spikelet.

The genetic material of crested wheatgrass Agropyron cristatum is also beneficial for yield components. Addition lines with chromosome 6P of Ag. cristatum and, particularly, substitution lines 6P/6D show high productive tillering and significantly greater grain numbers in spikes and spikelets: up to 4.5 grains per spikelet (Wu et al., 2006; Han H. et al., 2014). It has been inferred that chromosome 6P houses genes controlling the numbers of florets and grains in a spike and spikelet (Wu et al., 2006). Conceivably, chromosome 6Agi2 of Th. intermedium bears gene(s) controlling the synchronous metameric growth of spikelets in T10, whereas the additive manifestation of the trait ‘grain number per spikelet’ is observed in line 49-14 (T10 × S29), where up to six normal florets develop in a spikelet

## Conclusion

Sakuma et al., 2019) are means for improving wheat grain yield.

## Conflict of interest

The authors declare no conflict of interest.
